# Culture independent molecular analysis of bacterial communities in the mangrove sediment of Sundarban, India

**DOI:** 10.1186/1746-1448-6-1

**Published:** 2010-02-17

**Authors:** Abhrajyoti Ghosh, Nirmalya Dey, Amit Bera, Amit Tiwari, KB Sathyaniranjan, Kalyan Chakrabarti, Dhrubajyoti Chattopadhyay

**Affiliations:** 1 Department of Biochemistry and Department of Biotechnology, University of Calcutta, 35, Ballygunge Circular Road, Kolkata- 700019, West Bengal, India; 2 Department of Agricultural Chemistry & Soil Science, Institute of Agricultural Science, University of Calcutta, 35, Ballygunge Circular Road. Kolkata- 700019, West Bengal, India; 3 Current address: Max Planck Research Group "Molecular Biology of Archaea", Max Planck Institute for Terrestrial Microbiology, Karl-von-Frisch-Strasse, D-35043 Marburg, Germany; 4 Current address: Division of Nephrology Department of Medicine, 7703 Floyd Curl Drive, University of Texas Health Science Center at San Antonio, San Antonio, Texas 78229 3900, USA; 5 Current address: Institute of Molecular Cancer Research, University of Zurich, 17 K 28, Winterthurerstrasse 190, Zurich-8057, Switzerland

## Abstract

**Background:**

Sundarban is the world's largest coastal sediment comprising of mangrove forest which covers about one million hectares in the south-eastern parts of India and southern parts of Bangladesh. The microbial diversity in this sediment is largely unknown till date. In the present study an attempt has been made to understand the microbial diversity in this sediment using a cultivation-independent molecular approach.

**Results:**

Two 16 S rRNA gene libraries were constructed and partial sequencing of the selected clones was carried out to identify bacterial strains present in the sediment. Phylogenetic analysis of partially sequenced 16 S rRNA gene sequences revealed the diversity of bacterial strains in the Sundarban sediment. At least 8 different bacterial phyla were detected. The major divisions of detected bacterial phyla were Proteobacteria (alpha, beta, gamma, and delta), Flexibacteria (CFB group), Actinobacteria, Acidobacteria, Chloroflexi, Firmicutes, Planctomycetes and Gammatimonadates.

**Conclusion:**

The gammaproteobacteria were found to be the most abundant bacterial group in Sundarban sediment. Many clones showed similarity with previously reported bacterial lineages recovered from various marine sediments. The present study indicates a probable hydrocarbon and oil contamination in this sediment. In the present study, a number of clones were identified that have shown similarity with bacterial clones or isolates responsible for the maintenance of the S-cycle in the saline environment.

## Background

The majority (60-70%) of the world tropical and subtropical coastlines are covered with mangrove ecosystems. Mangroves are known to be highly productive ecosystems and have immense ecological values. They protect and stabilize the costal zones, nourish and nurture the coastal water with nutrients. They play important role as the feeding and breeding areas of many organisms including plants, animals and micro-organisms. The microbial community in the mangrove sediment is strongly influenced by bio-geographical, anthropological and ecological properties. These properties include food web in the ecosystem, nutrient cycling and the presence of organic and inorganic matters.

During the past decade, the development of molecular techniques using nucleic acids has led to many new findings in the studies of microbial ecology [1]. As a basic approach to clarify the microbial communities, 16S rRNA genes are amplified by PCR from nucleic acids extracted from environmental samples, and then the PCR products are cloned and sequenced. This approach can avoid the limitation of the traditional culturing techniques for assessing the microbial diversity in the natural environments.

Both the sediment and soil probably represent some of the most complex microbial habitats on the Earth. There may be several thousand species of bacteria in 1 g of soil [2]. To study the genetic diversity and to analyse the members of mixed microbial populations are the two most important steps in microbial community studies. However, little research has been done on microbial diversity in marine sediments, and little information is currently available [3].

Mangrove ecosystems are in general nutrient-deficient, especially of nitrogen and phosphorus [4-8]. In spite of this, mangroves are highly productive. Microbial activity is responsible for major nutrient transformations within a mangrove ecosystem [4,9]. In tropical mangroves, bacteria and fungi constitute 91% of the total microbial biomass, whereas algae and protozoa represent only 7% and 2%, respectively [10]. Bacteria are responsible for most of the carbon flux in tropical mangrove sediments. They process most of the energy flow and nutrients, and act as a carbon sink. For example, in semiarid mangrove ecosystems on the Indus river in Pakistan, bacteria were attached to the sediment particles and processed most of the ecosystem nutrients [11].

Several studies have shown the uniqueness of mangrove sediments with respect to their microbial composition [3,11-15]. Studies on microbial diversity in the mangrove sediments are important to understand the process of biogeochemical cycling and pollutants removal [16].

Sundarban is world's largest coastal wetland comprising of mangrove forest which covers about one million hectares in the delta of the rivers Ganga, Bramhaputra, and Meghna [17]. This mangrove region is shared between Bangladesh (~ 60%) and India (~ 40%). The area experiences a subtropical monsoon climate with the annual rainfall of about 1600-1800 mm and several cyclonic storms. The dynamics of this region is mainly maintained by sedimentations from all the three major rivers. Both the spatial and temporal influences have been demonstrated on the salinity in this region. The biodiversity of Sundarban includes numerous species of phytoplankton, zooplankton, micro-organisms, benthic invertebrates, molluscs, amphibians and mammals [17]. About 350 species of vascular plants, 250 species of fishes, and 300 species of birds are reported in Sundarban region [17]. Little work has been carried out on the microbial diversity in the Sundarban sediments.

This paper describes the culture independent microbial diversity analysis of the sediment sample from one of the famous islands of Sundarban, Netidhopani. Our molecular phylogenetic analysis reveals the occurrence of the bacterial 16S rRNA gene sequences that are unique and sequences that are previously reported in other mangrove sediments. Microbial community structure analysis can provide a better understanding about the microbial population and their interactions in a defined geographical region. Moreover such data are important with respect to our understanding of mangrove ecosystem processes and the role of micro-organisms in maintaining these processes [18].

The present results considerably extend our understanding on microbial diversity in Sundarban sediments. Furthermore, these results will open a new door towards understanding microbial diversity in the largest mangrove sediment in the world.

## Methods

### Site Selection

Sediment samples were collected between 1^st ^to 10^th ^November' 2006 from one of the composite islands of Sundarban, Netidhopani (21°55'13" N, 88°44'46" E). This area is inundated with sea water about every twelve hours. Soil was collected after recessation when the land was exposed. This soil was highly saline with Ec_e _12.6 dSm^-1^.

### Soil Sampling, Analyses, and Site Climate

Soil samples were collected from the top 15 cm of the five different sites on the island and brought to the laboratory in properly labelled, autoclaved, and sealed polythene bags on ice. All the soil analyses were carried out in the Department of Biotechnology, University of Calcutta. Microbiological and biochemical analyses were performed with the field moist soils. Physico-chemical analysis was carried out with air-dried soil samples. The soil pH was measured in 1:2.5 soil water suspensions. The Ec_e _and ionic composition of soil saturation extract were measured following the method described in United States Department of Agriculture (USDA), 1954 [19]. The organic C (OC) and total N (TN) were measured by the methods proposed by Nelson & Sommers 1982 and Black, 1965 [4,20] respectively. Textural composition (i.e., determination of sand, silt and clay) was determined by International Pipette Method as described previously [21]. The soil sample contained around 53.5% sand, 23.32% silt, 28% clay, 4.3% organic matter, and 0.395% total N (Average values of the triplicate analysis). The soil pH was found to be alkaline and it was 8.1. Prior to the total DNA isolation, all the soil samples (five samples were collected from different sites of the island) were mixed to homogeneity to make a single composite sample for DNA isolation.

### Isolation of total soil DNA

Total soil (sediment) DNA was isolated using a modified CTAB-SDS based DNA extraction technique [22]. Soil sample of 5 g was mixed with 13.5 ml of the DNA extraction buffer [100 mM Tris-HCl (pH 8.0), 100 mM sodium-EDTA (pH 8.0), 100 mM sodium phosphate buffer (pH 8.0), 1.5 M NaCl, 1% CTAB] and 100 μl of proteinase K (10 mg/ml) in oakridge tube by horizontal shaking at 200 rpm for 30 min at 37°C. After the shaking treatment, 1.5 ml of 20% SDS was added, and then the sample was incubated at 65°C water bath for two hours with gentle end-over-end inversions every 15-20 min. The supernatant was collected after centrifugation at 6000 × g for 10 min at room temperature into 50 ml centrifuge tube. The soil pellet was extracted two more times by adding 4.5 ml of the extraction buffer and 0.5 ml of 20% SDS; vortexing for 10 s, incubating at 65°C for 10 min, and centrifuging as before. Supernatants from three cycles of extractions were combined and mixed with an equal volume of chloroform-isoamyl alcohol (24:1 v/v). The aqueous phase was recovered by centrifugation and total nucleic acid was precipitated with 0.6 volume of isopropanol at room temperature for 1 h. The pellet of crude nucleic acid was obtained by centrifugation at 16,000 × g for 20 min at room temperature, washed twice with cold 70% ethanol, and resuspended in sterile deionised water to give a final volume of 250 μl. RNase (10 mg/ml) treatment followed by phenol extraction and the re-precipitation was carried out prior to PCR amplification of 16S rRNA gene sequences.

### PCR amplification and partial 16S rRNA gene library construction

Partial amplification of the 16S rRNA gene was performed with the thermal cycler ABI 2700 (ABI, Foster City, USA). The PCR of the 16S rRNA gene sequence from the total soil DNA was conducted in a final volume of 50 μl. The reaction mixture included 20-50 ng of isolated total soil DNA, 2 U taq polymerase (Recombinant, Cat. No. SKU# 10342-020, Invitrogen, Germany), 1× PCR buffer with 1.5 mM MgCl_2_, 200 mM each dNTP, and 10 pmol of each primer (IDT, USA). The primers were chosen to amplify a 977-bp segment of 16S rRNA gene spanning V3-V9 region to construct the first library. In the first library (D16S_pMOS library) construction, forward primer used was 515F (5'-3') GTGCCAGCAGCCGCGGTAA and the reverse primer was 1492R (5'-3') TACGGYTACCTTGTTACGACTT [23]. This pair of primers was chosen to amplify both the bacterial and archaeal 16S rRNA gene sequences in the total soil DNA. Before amplification cycle DNA was denatured for 2 min at 94°C and after amplification an extension step (7 min at 72°C) was performed. The cycling parameters consisted of 28 cycles at: denaturation at 94°C for 30 sec, primer annealing at 45°C for 1 min, extension at 72°C for 1 min. The samples were held at 4°C until separated electrophoretically in a 2% agarose gel in 0.5 × Tris-Borate-EDTA buffers and visualized using ethidium bromide under ultraviolet illumination. All the amplified PCR products were agarose-gel-eluted using Qiagen gel elution kit.

Since the proteobacterial population has previously been reported to be quite abundant in other saline sediments [3,24-26] a second library was also constructed using the proteobacteria specific universal SSU primer set [27], uni-forward (5'-3') TGCCAGCAGCCGCGGTA and uni-reverse (5'-3') GACGGGCGGTGTGTACAA, to screen for proteobacterial population in Sundarban sediments. The amplification cycle was as follows; initial denaturation for 5 min at 94°C, followed by 35 cycles at: 30 sec denaturation at 94°C, 1 min for primer annealing at 50°C, and 1 min of extension at 72°C. After the amplification a final extension step (7 min at 72°C) was performed. The samples were held at 4°C until analysis by agarose gel electrophoresis followed by elution using the Qiagen gel elution kit.

Two partial 16S rRNA gene libraries were constructed using gel eluted amplified PCR products (515F/1492R amplicon and uni-for/uni-rev amplicon) and pMOS-Blue vector (Pharmacia). The gel eluted PCR products were ligated to pMOS-Blue vector and then transformed into competent *Escherichia coli *XL1-Blue. The clones were screened for α-complementation by using X-Gal (5-bromo-4-chloro-3-indolyl-β-D-galactopyranoside) and IPTG (isopropyl-β-D thiogalactoside). All the positive clones were confirmed by PCR amplification and restriction digestion. All the positive clones were stored as glycerol stock at -70°C freezer.

### Sequencing of the 16S rRNA gene Fragment

The sequencing of the partial 16S rRNA gene fragments in each of the recombinant plasmids was performed in ABI Prism 3100 automated DNA sequencer (Applied Biosystem, Foster City, California, USA) with the single primer 515F for D16S_pMOS library and uni-for for DUni_pMOS library, respectively. The sequencing reaction was performed using 5 pmoles 515F/uni-for primer and the Big Dye Terminator V3.1 sequencing kit as per manufacturer's protocol. The sequencing reaction conditions were as follows: 96°C for 10 sec, 50°C for 10 sec, and 60°C for 4 min for 25 cycles. After the sequencing PCR, the products (10 μl) were treated with 2 μl of 125 mM Na-EDTA, pH 8.0, and then precipitated using 2 μl of 3 M NaOAc (pH 4.6) and 50 μl absolute ethanol for 20 minutes at room temperature. The DNA was recovered by centrifugation (13,000 rpm for 30 min at 20°C), washed with 70% EtOH, dried, and resuspended in 15 μl Hi Di formamide (Applied Biosystems, Foster City, California, USA). Sequencing was performed in the ABI Prism 3100 Genetic Analyzer. Raw sequences were edited and assembled using the Auto Assembler program (V5.2). All the sequences were used to identify the bacteria with the help of the BLASTn program http://www.ncbi.nlm.nih.gov/BLAST, and all the sequences were submitted to GenBank. The multiple sequence alignment was performed using CLUSTAL-W software package http://www.ebi.ac.uk/Tools/clustalw2/index.html.

### Blast Search & Phylogenetic Analysis

The partial 16S rRNA gene sequences of the clones were compared with those available in the public databases. Identification to the species level was determined as a 16S rRNA gene sequence similarity of ≥ 97% with that of the prototype strain sequence in the GenBank. Sequence alignment and comparison was performed using the multiple sequence alignment program CLUSTALX (v 1.83) [28], with default parameters and the data converted to PHYLIP format. Minor modifications in the alignment were made using the BIOEDIT sequence editor. Rooted and unrooted phylogenetic trees were constructed using neighbor-joining (NJ) method and the TREEVIEW program for display of phylogenetic relationship [29]. Bootstrap analysis was performed as described by Felsenstein in 1985 [30] on 1000 random samples taken from the multiple alignments; analysis was done using the ClustalX programs.

### Nucleotide sequence accession numbers

The 16S rRNA gene sequences reported in this study was submitted to the GenBank database under accession numbers EU939923-EU939972 (D16S_pMOS library) and EU999048-EU999127 (Duni_pMOS library).

## Results and discussion

### DNA extraction, Library construction and sequencing analysis

**Figure 1:**
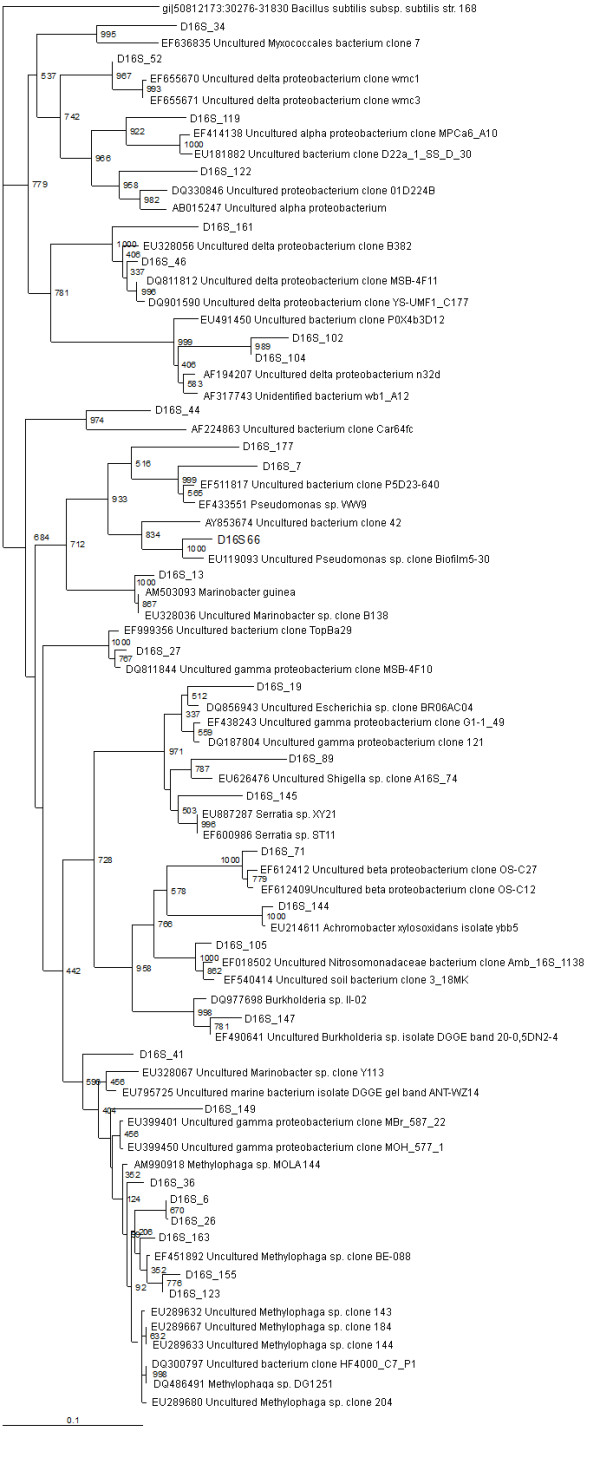
**16S rRNA gene tree showing positions of proteobacterial sequences in D16S_pMOS library including the reference sequences retrieved from GenBank**. 16S rRNA gene sequence of *Bacillus subtilis *168 is used to assign an out-group species.

**Figure 2:**
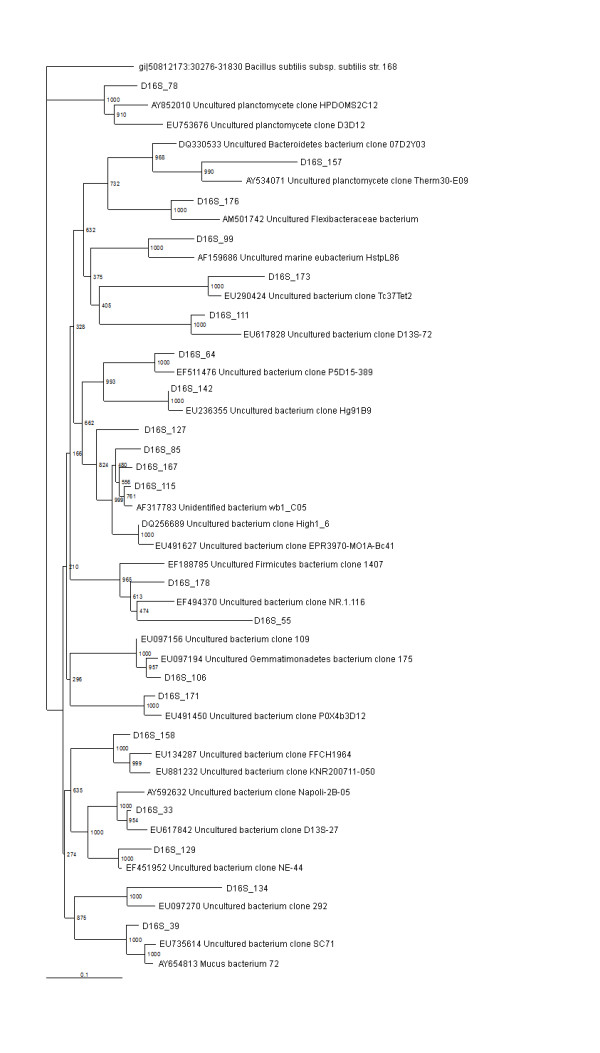
**16S rRNA gene tree showing positions of non-proteobacterial sequences (Flexibacteria, Planctomycetes, Firmicutes, Gemmatimonadates, unidentified/uncultured) in D16S_pMOS library including the reference sequences retrieved from GenBank**. 16S rRNA gene sequence of *Bacillus subtilis *168 is used to assign an out-group species.

**Figure 3:**
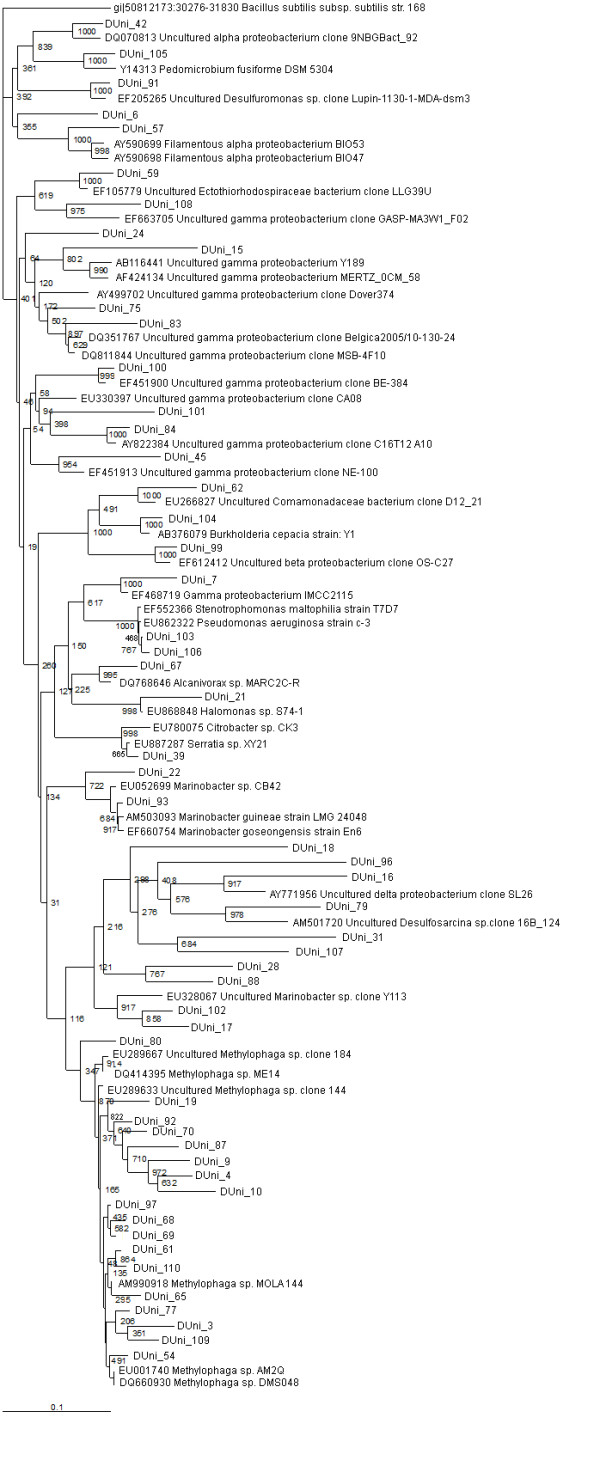
**16S rRNA gene tree showing positions of proteobacterial sequences in DUni_pMOS library including the reference sequences retrieved from GenBank**. 16S rRNA gene sequence of *Bacillus subtilis *168 is used to assign an out-group species.

**Figure 4:**
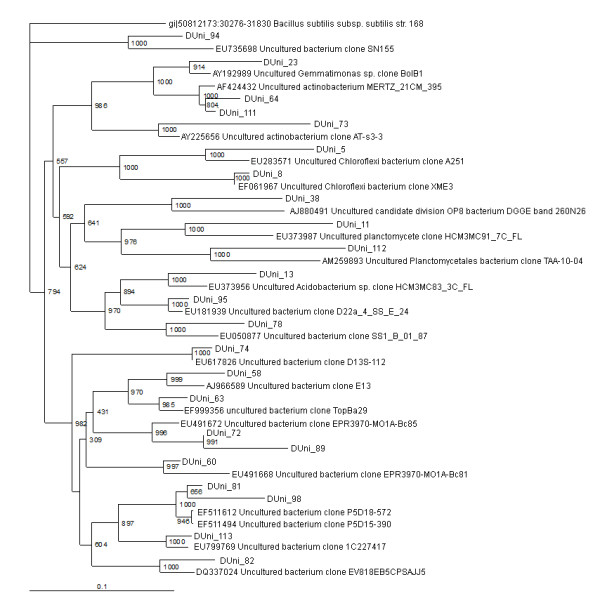
**16S rRNA gene tree showing positions of non-proteobacterial sequences (Actinobacteria, Planctomycetes, Chloroflexi, Acidobacteria, Gemmatimonadates, unidentified/uncultured) in D16S_pMOS library including the reference sequences retrieved from GenBank**. 16S rRNA gene sequence of *Bacillus subtilis *168 is used to assign an out-group species.

The total DNA was extracted from the sediment of Netidhopani, Sundarban, using modified CTAB-SDS based DNA isolation technique. Two partial 16S rRNA gene clone libraries were established from the PCR amplified partial 16S rRNA gene sequences using 515F/1492R and uni-for/uni-rev primer sets, respectively. The recombinant clones in the libraries were selected based on α-complementation (blue-white screening) technique and also confirmed by the re-PCR analysis and restriction enzyme digestion. Our sequencing analysis included 85 clones from D16S_pMOS library and 110 clones from DUni_pMOS library. All the sequenced clones were screened for sequences that repeat more than once in the library. Our final analysis included 50 clones from D16S_pMOS library and 80 clones from DUni_pMOS library, respectively (Table1) (Figures 1, 2, 3 and 4). *Methylophaga spp. *was found to be abundant in both the libraries. We also found four non bacterial chloroplastic DNA in recombinant clones from the two libraries. Although, the primer pair 515F & 1492R could amplify both bacterial and archaeal 16S rRNA gene sequences, we did not get any archaeal sequence in our library (D16S_pMOS). This was probably because of the limitation in our total DNA extraction protocol and low primer specificities towards the archaeal 16S rRNA gene sequences. Our phylogenetic analysis revealed that 130 bacterial clones (50 clones from D16S_pMOS library and 80 clones from DUni_pMOS library) fell into 8 major phyla of the bacterial domain: Proteobacteria (Alpha-, Beta-, Gamma-, and Delta-), the Cytophaga-Flexibacter-Bacteroides (CFB) group, Actinobacteria, Chloroflexi, Firmicutes, Gemmatimonadetes, Acidobacteria group, and Planctomycetes (Table 1).

**Table 1:** Summary of the 16S rRNA gene sequences identified in the D16S_pMOS and DUni_pMOS clone libraries

Bacterial division	Number of sequence types	Number of sequence types in D16S_pMOS library (%)	Number of sequence types in DUni_pMOS library (%)	Number of total clones (%)	**Sequence similarity to the closest relatives**^c^**(%)**
Proteobacteria	86	29 (58%)^a^	57 (71%)^a^	66.1	83-100

Alpha	6	1 (3.5%)^b^	5 (8.7%)^b^	4.6	87-96

Beta	7	4 (14%)^b^	3 (5.2%)^b^	5.4	92-99

Gamma	61	17 (59%)^b^	44 (77%)^b^	47	86-100

Delta	10	6 (21%)^b^	4 (7%)^b^	7.7	83-99

Unassigned proteobacteria	2	1 (2%)^b^	1 (1.25%)^b^	1.5	91-94

CFB group	1	1 (2%)^a^	0	0.8	93

Actinobacteria	2	0	2 (2.5%)^a^	1.53	87-98

Planctomycetes	4	2 (4%)^a^	2 (2.5%)^a^	3	86-92

Firmicutes	1	1 (2%)^a^	0	0.8	92

Chloroflexi	2	0	2 (2.5%)^a^	1.53	89-98

Gemmatimonadates	2	1(2%)^a^	1 (1.25%)^a^	0.75	94-96

Acidobacteria	1	0	1 (1.25%)^a^	0.8	91

Marie eubacterium	1	1 (2%)^a^	0	0.8	89

Bacterial candidate division OP8	1	0	1 (1.25%)^a^	0.8	87

Uncultured	24	11 (22%)^a^	13 (16%)^a^	18.5	86-99

Unidentified	5	4 (8%)^a^	1 (1.25%)^a^	3.9	91-98

Total	130	50	80	--	83-100

"a" % of clones among all the 130 clones selected from D16S_pMOS and DUni_pMOS libraries

"b" % of clones among the proteobacterial clones under respective library

"c" Closest relatives as determined by the BLAST analysis

### Proteobacteria

A total of 86 clones represented by 29 sequence types from D16S_pMOS library and 57 sequence types from DUni_pMOS library were identified as proteobacterial in origin by sequencing analysis (Table 1) (Figure 3). Most of the proteobacterial sequence types from both the libraries were similar to previously described isolates or metagenomic clones from coastal marine sediments or waters [GenBank description, sequences that were submitted to GenBank but yet to be published in scientific journals]. In D16S_pMOS library 29 proteobacterial sequence types accounted for 58% of the gene library and in DUni_pMOS library 57 proteobacterial sequence types accounted for 71% of the gene library (Figure 1).

#### Gammaproteobacteria

The gammaproteobacteria represented the most abundant proteobacterial subdivision (59% and 77% among the proteobacterial sequence types in D16S_pMOS and DUni_pMOS libraries, respectively) (Table 1). The most abundant sequence type in both the libraries showed similarity to *Methylophaga*, indicating a strong involvement of these bacterial species in the maintenance of the biogeochemical cycle in Sundarban sediment. A number of gammaproteobacterial sequence types showed similarity to organisms involved in the S-cycle (DUni_9, DUni_15, DUni_68, DUni_77, and DUni_91). A number of the gammaproteobacterial clones showed sequence similarity to the oil (D16S_41, DUni_17, DUni_102) and hydrocarbon (DUni_3, DUni_9, DUni_18, DUni_22, DUni_54, DUni_61, DUni_62, DUni_67, DUni_68, DUni_69, DUni_77, DUni_84, DUni_90, DUni_103, DUni_109, and DUni_110) degrading bacterial populations reported in different soil system [GenBank description, [31-33]]. Three sequence types (D16S_89, D16S_145 and DUni_83) have shown similarity to previously extracted sequences from the heavy metal contaminated soil or sediments [GenBank description, [34]]. Previous studies on Sundarban region revealed the contamination of hydrocarbon, petroleum and heavy metals in the soil [16,35-38]. The microbial composition further indicates the previous observations and a probable possibility of bio-conversion of those contaminating substances in this soil area.

#### Betaproteobacteria

14% and 5% of the proteobacterial sequence types were found to be betaproteobacterial from D16S_pMOS and DUni_pMOS libraries respectively (Table 1). In the D16S_pMOS library, the predominating clone (D16S_105) (three clones in the library) displayed a sequence similarity of 96% to its closest relative Amb_16S_1138, a betaproteobacterial clone previously recovered from trembling aspen [GenBank description]. In the DUni_pMOS library, among all the clones, DUni_99 showed 97% similarity to an uncultured betaproteobacterial clone OS-C27, recovered from the abandoned semiarid lead-Zn mine tailing site [GenBank description, [39]]; DUni_62 showed 92% similarity to D12-21, a betaproteobacterial clone recovered from a tar oil contaminated plume [GenBank description]; and finally the third clone DUni_104 showed 97% similarity to *Burkholderia cepacia *strain Y1 isolated from oil polluted soil.

#### Alphaproteobacteria

The six alphaproteobacterial sequence types detected in the two libraries comprised 3.5% and 8.7% of the total proteobacterial sequence types in D16S_pMOS and DUni_pMOS respectively (Table 1). In the D16S_pMOS library, the clone D16S_119 was the only sequence type and that showed 95% similarity to MPCa6_A10, an alphaproteobacterial clone recovered from wild and captive sponge *Microciona prolifera *in the Chesapeake Bay [GenBank description]. Five different alphaproteobacterial clones were obtained in DUni_pMOS library. The most predominating sequence type was DUni_6, which showed 87% similarity to MERTZ_OCM_210, an alphaproteobacterial clone reported within the Antarctic continental shelf sediment [GenBank description]. Among the other clones, sequence similarity revealed that they have shown identity to clones from the Seafloor Basalts from East Pacific Rise and the Juan de Fuca Ridge (DUni_42), industrial waste water treatment plants (DUni_56, DUni_57) [40] and *Pedomicrobium fusiform *DSM 5304 (DUni_105) [GenBank description, [41]].

#### Deltaproteobacteria

2.1% and 7% of the cloned sequence types have shown similarity to the deltaproteobacterial sequences in the database from D16S_pMOS and DUni_pMOS libraries, respectively (Table 1). In the D16S_pMOS library, all the deltaproteobacterial clones showed identity to organisms or clones recently described in Mangrove sediments. The sequence type D16S_52 was found to show 99% similarity with sulphate reducing bacterial strains [GenBank description]. In the DUni_pMOS library, clones DUni_79 and DUni_91 showed similarity with *Desulfosarcina spp. *and *Desulfuromonas spp. *respectively. These genera members have well-known metabolic features and involved in maintenance of S-cycle in soil. Probably the deltaproteobacterial strains in Sundarban sediments are largely involved in contributing towards the maintenance of sulphur cycle by sulphate reduction.

In addition to defined identities, two clones each from the D16S_pMOS and DUni_pMOS libraries showed only their proteobacterial identity. The clone D16S_122 showed similarity to uncultured proteobacterial clone 01D224B, which was described previously in the Guerrero Negro hyper saline microbial mat [GenBank description]. The clone DUni_55 showed identity to uncultured proteobacterial clone SIMO-1762, recovered from the salt marsh [GenBank description].

In both the libraries, many of the proteobacterial sequence types were found to show phylogenetic similarity with the strains (isolates) or clones recently described in marine sediments and waters, and were involved in S- or N-cycles, e.g., *Methylophaga spp. *DMS044, *Methylophaga spp. *DMS048, Uncultured gammaproteobacterium Y189 [42], Uncultured deltaproteobacterium wmc3 [GenBank description, EF655671], Uncultured *Nitrosomonadaceae *clone Amb_16S_1138 [GenBank description, EF018502], Uncultured *Desulfuromonas spp. *clone Lupin-1130-1-MDA-dsm3 [GenBank description, EF205265]. A large number of sequence types were also found to be phylogenetically similar to oil degradation associated microbes, e.g., Uncultured *Marinobacter spp. *Clone Y113 [GenBank description, EU328067], Uncultured gammaproteobacterium clone Y168 [GenBank description, EU328083]. There was also clone (DUni_67) identical to previously identified PAH degrading bacterial isolates [GenBank description]. Moreover in our libraries, we found many 16S rRNA gene sequences (D16S_6, D16S_66, D16S_89, D16S_106, D16S_123, D16S_134, D16S_155, D16S_163, DUni_4, DUni_19, DUni_45, DUni_65, and DUni_100) were similar to metagenomic clones or isolates reported in other studies from India [GenBank description, [7]]. In Sundarban sediment, compounds like polybrominated diphenyl ether (PBDE) and other hydrocarbons have been reported by different groups [35,36,38]. Moreover, in this sediment high concentration of heavy metal has been reported previously [37]. Furthermore, there are reports on the isolation of oil (petroleum) degrading bacterial strains from Sundarban sediment [16]. All this analytical and microbiological evidences further support our findings of different bacterial species related to bacterial clones or strains previously reported in hydrocarbon, oil, and heavy metal contaminated soils and sediments.

### Cytophaga-Flexibacteria-Bacteriodes

A single sequence type, representing a total of six clones and accounting for 2% of the D16S_pMOS library, was found to cluster with the CFB group (Table 1) (Figure 2). The representing clone D16S_176 showed similarity with the *Flexibacteraceae*, bacterium recently reported from Venice Lagoon anoxic sediments [GenBank description]. No clone related to the CFB group was detected in DUni_pMOS library.

### Chloroflexi

Two clones in DUni_pMOS library showed similarity with the Chloroflexi (Table 1) (Figure 4). The clone DUni_5 showed sequence similarity with A251, an uncultured *Chloroflexi *[GenBank description]. Another clone DUni_8 showed similarity with XME3, an uncultured Chloroflexi, recently reported in mangrove sediment of Xiamen, China [GenBank description].

### Planctomycetes

Two sequence types, representing 6 clones in D16S_pMOS library and two sequence types, representing 3 clones in DUni_pMOS library, were found to group within the Planctomycetes (Table 1) (Figures 2 and 4). In D16S_pMOS library, the clone D16S_78 showed similarity to D3D12, an uncultured planctomycete clone recovered from the fresh water stromatolites from the Ruidera Pools Natural Park, Spain [GenBank description]. The clone D16S_157 was found to be identical to Therm30-E09, an uncultured planctomycete clone reported in the sediment of the Eastern Mediterranean Sea [12]. In DUni_pMOS library, the clone DUni_112 showed similarity to TAA-10-04, an uncultured Planctomycete clone recovered as the phage associated bacterium and the clone DUni_11 was found to show similarity with HCM3MC91_7C_FL, the planctomycete clone recovered from Eastern Mediterranean Sea [GenBank description].

### Other bacterial lineages

A total of six clones, represented by two sequence types and accounted for 2.5% of the DUni_pMOS library were found to group within the Actinobacteria (Table 1). Two representative sequence types, DUni_64 and DUni_73 showed similarities with uncultured actinobacteria clone MERTZ_21CM_395 [43] and uncultured actinobacterial clone AT-s3-3 [43], respectively (Figures 2 and 4).

Two clones each from the two libraries were grouped within the Gemmatimonadetes (Table 1), a recently discovered bacterial phylum [44]. The clone D16S_106 showed 96% similarity to Gemmatimonadetes bacterial clone 175, recovered from soil sample from radish rich area of Jaunpur, Uttarpradesh, India [GenBank description]. The other clone DUni_23 was found to be 94% identical to Gemmatimonadetes bacterial clone BolB1, reported recently as a member of phylogenetic division OP11 [45].

A single clone, DUni_38 was found to be identical to recently reported uncultured candidate division OP8 [GenBank description, [46]]. A single clone, D16S_178 was found to show similarity with the uncultured Firmicutes bacterium clone 1407, which was previously reported in the Altamira Cave [GenBank description]. Among the clones in the libraries, the clone DUni_13 showed similarity to HCM3MC83_3C-FL, an *Acidobacterium spp. *clone recently recovered from the sediment of Eastern Mediterranean Sea [GenBank description].

Other than known bacterial taxa, 22% and 16% of the clones from D16S_pMOS and DUni_pMOS libraries [26], were clustered within uncultured bacterial group (Table 1) (Figures 2 and 4). Most of the related uncultured bacterial clones from the blast search analysis revealed that they were reported from either marine sediments or from sea waters. In our library of clones, we also detected unidentified bacterial strains (four in D16S_pMOS and one in DUni_pMOS libraries). The only clone, D16S_99 showed identity to marine eubacterium HstpL86, previously described in the leaves of sea grass *Halophila stipulacea*.

## Conclusions

In the present study, 16S rRNA gene clone library based analysis was performed on the world's largest mangrove ecosystem, Sundarban sediment, for the first time. Even no culture based analysis of the bacterial community is yet reported from this mangrove ecosystem. The present analysis revealed that the Sundarban sediment possesses diverse bacterial population. At least 8 major phyla of the bacterial domain were detected in this sediment. Previous studies on bacterial diversity analysis reported five to thirteen major lineages in sediments collected from a variety of coastal marine environments [3,15,24,25,42].

Sequencing analysis of the clones revealed the dominance of gammaproteobacterial sequences in both the libraries. Majority of the gammaproteobacterial clones resembled sequences recovered from oil and hydrocarbon rich marine sediments. This probably goes with the previous reports on Sundarban sediment where people have shown that in this sediment different hydrocarbons are present at high concentrations [35,36,38]. Moreover, a number of cultivable bacterial strains, which were capable of degrading petroleum, have been isolated from this sediment [16].

In the present report, a number of gammaproteobacterial clones were found to show similarity towards bacterial clones or isolates involved in sulfur cycling. Similar results were reported previously [42], while analysing coastal marine sediment beneath area of intensive shellfish aquaculture. Sulfur-oxidising bacterial strains are found to play an important role in detoxification of sulphide in marine sediments. Sulfur-reducing bacterial community instead is important in organic carbon oxidation in marine sediments and this observation is supported by the fact that sulphate is one of the main electron acceptors present in these environments. A number of gammaproteobacteria in the present study were found to show similarity to isolates or clones related to bioconversion of S-containing organic molecules (S-oxidisers). This interesting observation is supported by recent investigations, where it has been shown that the reduction of sulphate may be an important pathway of organic matter mineralization in organic rich deposits typical of mangrove forests. Furthermore, most of the identified deltaproteobacterial clones from the two libraries showed similarity to the sulfur and sulphate reducing bacteria recovered from a variety of marine sediments. Analysis of only 130 clones might not be enough to cover the whole picture of S-cycle but it could provide a little insight about what is happening in Sundarban sediment. In marine ecosystems, S-cycle has proved to be the important biogeochemical factor that dictates the flow of electrons along the biological systems under such an anaerobic condition. Identification of sulfur- oxidising and sulphur and sulphate reducing bacterial clones refer to the anaerobic condition in this sediment and a possible maintenance of the biogeochemical cycle in Sundarban sediment.

The evidence of the presence of hydrocarbons [35,36,38] in this sediment supports the finding of a comparatively lower number of alphaproteobacterial clones in Sundarban sediment. The special difference in the productivity of the water columns is probably enhances the reason for this observation in this sediment. Previous observations of Horner-devine *et.al.*, 2003 [47] showed the dependence of alphaproteobacteria richness on the productivity levels in aquatic ecosystem. Furthermore, identification of the clones associated with hydrocarbon/oil degradation probably confirms the reason for the lower abundance of alphaproteobacteria in Sundarban sediment.

In our study, epsilonproteobacteria are absent in both the libraries. It has been well documented that epsilonproteobacteria were absent or scarce in other clone libraries of coastal marine sediments [3,15,42]. Although, some reports have described the presence of epsilonbacteria in the library of clones made from marine sediments [24,48].

Identification of Gemmatimonades in our clone libraries was interesting and probably the first report of recovery of this phylum from mangrove sediment.

In conclusion, the Sundarban surface sediment harboured a phylogenetically diverse population of organisms from bacterial domain. At least 8 major phyla have been recovered from Sundarban sediment. The proteobacteria, especially the gammaproteobacteria were found to be abundant in both the libraries. While some of the 16S rRNA gene sequence types detected were related to genera or taxa that were classically identified in Sundarban sediment and correlated to a defined functional arena; many were derived from uncultured/unidentified taxa. Previous studies have shown that the primer pair uni-for/uni-rev was good for proteobacteria. As in marine system, proteobacterial communities are major; this pair of primers was employed in the present study. Further studies are necessary to understand the bacterial diversity in more details. This is the first report describing the bacterial diversity in Sundarban sediment. We feel that the present study has obtained a fundamental insight into the major bacterial populations in Sundarban sediment. This study will definitely open a new era in understanding the microbial diversity in Sundarban.

## Competing interests

The authors declare that they have no competing interests.

## Authors' contributions

AG designed and conducted the experiments in consultation with DJC, analyzed the data and drafted the manuscript. ND helped with the library constructions, screening and sequencing analysis. AB participated in sequencing analysis, sequence alignment and phylogenetic analysis. AT helped with the screening of the libraries and sequence alignment. SB helped with the screening of the libraries. KC helped with the sampling, soil analyses and interpretation. DJC was involved in the acquisition of funding, contributed to the concept, experimental design, analysis of the data and revising the manuscript. All authors read and approved the final manuscript.
